# Family‐Centred Care for Children and Adolescents Living With HIV in Sub‐Saharan Africa: A Scoping Review

**DOI:** 10.1002/jia2.70098

**Published:** 2026-05-20

**Authors:** Tawanda Makusha, Zinhle Ngubo, Janet Seeley, Moherndran Archary

**Affiliations:** ^1^ Africa Health Research Institute Durban South Africa; ^2^ Population and Development Studies University of KwaZulu‐Natal Durban South Africa; ^3^ Division of Medicine University College London London UK; ^4^ Department of Global Health and Development London School of Hygiene and Tropical Medicine London UK; ^5^ Medical Research Council/Uganda Virus Research Institute and London School of Hygiene & Tropical Medicine Uganda Research Unit Entebbe Uganda; ^6^ School of Nursing and Public Health Discipline of Public Health Medicine, University of KwaZulu‐Natal Durban South Africa; ^7^ Nelson Mandela School of Medicine University of KwaZulu‐Natal Durban South Africa; ^8^ Department of Health KwaZulu‐Natal South Africa

**Keywords:** child and adolescent HIV care, family‐centred care, implementation models, psychosocial support, retention in care, sub‐Saharan Africa

## Abstract

**Introduction:**

Children and adolescents living with HIV in sub‐Saharan Africa (SSA) continue to face significant barriers to diagnosis, treatment adherence, retention in care and psychosocial support, despite progress in prevention and treatment coverage. Family‐centred care (FCC) has emerged as a promising model that positions families—not just individual children—as the central unit of care, with potential to improve clinical and psychosocial outcomes while addressing persistent gaps in health system responsiveness.

**Methods:**

Our scoping review synthesized evidence on FCC implementation strategies and outcomes for children and adolescents living with HIV in SSA, to inform the development of a contextually relevant FCC intervention. We followed Arksey and O'Malley's five‐stage framework and the PRISMA‐ScR guidelines. A comprehensive search of peer‐reviewed and grey literature published between 2000 and 2025 was conducted across multiple databases and organizational websites between May and July 2025. Fourteen studies met the inclusion criteria and were selected for analysis.

**Results:**

FCC was conceptualized as a multidimensional framework integrating clinical care, psychosocial support, caregiver engagement, treatment literacy and community‐based delivery. Implementation models ranged from facility‐based integrated services to community‐driven and hybrid approaches. Interventions commonly included antiretroviral therapy provision, family‐based testing, counselling and enhanced psychosocial wellbeing. Community‐based and decentralized models showed particular promise in improving retention and reducing structural barriers. However, gaps persist, including limited longitudinal data, underrepresentation of fathers and adolescent caregivers, narrow assumptions of nuclear family structures and a lack of standardized FCC frameworks.

**Discussion:**

Most FCC interventions were small‐scale and donor‐funded, raising concerns regarding sustainability and health‐system integration. Additionally, many studies assumed nuclear family structures, overlooking the role of extended and non‐biological caregivers common in African contexts. The limited engagement of diverse caregiving networks and inconsistent conceptualization of FCC constrain its broader applicability and scalability.

**Conclusions:**

FCC offers a compelling and contextually appropriate model for strengthening HIV care for children and adolescents in SSA. Advancing the FCC requires standardized frameworks, inclusive and context‐sensitive programme design, and integration into national health systems to ensure equitable and sustainable delivery of holistic HIV care.

## 1 Introduction

In 2024, approximately 86% of the 1.4 million children aged 0–14 living with HIV globally lived in sub‐Saharan Africa (SSA) [1, 2]. Despite significant progress in the prevention of mother‐to‐child transmission (PMTCT), with countries like Botswana and Namibia nearing elimination thresholds, an estimated 120,000 children acquired HIV, and 75,000 children died of AIDS‐related illnesses globally [3]. While antiretroviral therapy (ART) coverage has expanded, only 55% of children living with HIV were receiving ART in 2024, compared to 77% of adults [4]. Without timely intervention, half of untreated children with HIV die by age 2, and 80% do not survive past their fifth birthday [5].

Adolescents and young people represent another vulnerable group disproportionately affected by HIV in SSA. In 2023, an estimated 1.9 million adolescent girls and young women (AGYW) aged 15–24 were living with HIV globally, compared to 1.2 million adolescent boys and young men [6]. Of the 210,000 new HIV acquisitions among AGYW in 2023, 77% occurred in SSA, with nearly two‐thirds concentrated in eastern and southern Africa [6]. Adolescent girls in the region were six times more likely to be newly acquire HIV than their male counterparts [7]. This disparity is driven by a complex interplay of biomedical, socio‐economic and cultural factors. These include delayed diagnosis, limited access to child‐friendly ART formulations for young children and systemic barriers within healthcare systems, including weak linkages between maternal and child health services for young children [4], and stigma and discrimination for children and adolescents [8].

Family‐centred care (FCC) is increasingly recognized as a practical strategy in addressing child and adolescent HIV and AIDS, particularly in SSA [9, 10]. The siloed approach—where services for adults, children and pregnant women are delivered separately—has proved to be insufficient in addressing the complex, interconnected needs of families affected by HIV. In contrast, FCC offers a holistic and contextually grounded framework to address these challenges. Recognizing that HIV is experienced and managed within the context of interpersonal relationships, complex social dynamics and broader economic systems, FCC places families, not just the individual child, at the heart of the care process. This approach emphasizes shared decision‐making, collaborative treatment planning and the critical role of caregivers, families, and communities in supporting adherence, reducing stigma and long‐term health outcomes. FCC is particularly relevant in SSA contexts, where caregiving is often distributed across extended family networks, including mothers, fathers, grandparents, aunts, uncles and older siblings. The alignment of FCC with cultural norms enhances both clinical and social outcomes [11], integrating psychosocial support, caregiver empowerment and community engagement into the continuum of care [2, 5].

## 2 Methods

Our scoping review aimed to synthesize evidence on FCC implementation strategies and outcomes for children and adolescents living with HIV in SSA. We followed the five‐stage methodological framework proposed by Arksey and O'Malley [12], refined by Levac et al. [13] and aligned with the PRISMA‐ScR (Preferred Reporting Items for Systematic Reviews and Meta‐Analyses extension for Scoping Reviews) guidelines [14]. The five stages of the review process were: (1) identifying the research question; (2) identifying relevant studies; (3) study selection; (4) charting the data; and (5) collating, summarizing and reporting the results. As this study was a scoping review of published literature, no primary data were collected, and ethical approval was not required. A consent waiver was, therefore, not applicable. Since this review did not involve human participants or primary data collection, informed consent was not required.

### 2.1 Stage 1: Identifying the Research Questions

We used the population, concept and context framework to guide our research questions, which focused on children and adolescents (population), FCC (concept) and SSA (context). Our review questions were:

1. What are the core components and characteristics of FCC models for children and adolescents living with HIV in SSA?
2. What implementation strategies have been employed to deliver FCC for children and adolescents living with HIV in this region, and what contextual factors influence their effectiveness?
3. What outcomes appear to have been associated with FCC interventions for children and adolescents living with HIV in SSA?

### 2.2 Stage 2: Identifying the Relevant Studies—Search Strategy

The literature search and data extraction for this review were conducted between May and July 2025 and were limited to empirical studies published between 2000 and 2025 examining FCC for children and adolescents (birth to 18 years) living with HIV in SSA. Search terms included combinations of concepts related to family‐centred care, children living with HIV, outcomes and sub‐Saharan Africa (see Table 1). A comprehensive search was conducted across PubMed, MEDLINE, EBSCOhost, PsycINFO and PsycARTICLES, selected for their broad coverage of relevant peer‐reviewed literature. Grey literature sources were also searched, including International AIDS Society conference abstracts and websites of major organizations such as the World Health Organization (WHO), Joint United Nations Programme on HIV/AIDS (UNAIDS), United Nations Children's Fund (UNICEF) and United States Agency for International Development (USAID), and reference lists of included studies were hand‐searched to identify additional relevant publications.

**TABLE 1:** Search strategy, search terms and database adaptations.

Concept	Search terms (free text and synonyms)	Controlled vocabulary used (examples)	Field restrictions	Truncation/Boolean logic	Notes on database adaptation
Family‐centred care	“family‐centred care” OR “family‐centered care” OR “family‐focused care” OR “family‐centred approach” OR “family‐centered approach” OR “family‐based intervention*” OR “family support program*” OR “family support programme*” OR “caregiver support” OR “family involvement in care” OR “community‐based family support” OR “parenting intervention*” OR “family cohesion”	MeSH: Patient‐Centered Care; Family; Caregivers. PsycINFO Thesaurus: Family processes; Caregiving	Title/Abstract (and keywords where available)	OR used within concept; truncation (*) used for intervention/programme variations	Search terms adapted to database indexing; broader keywords used where controlled vocabulary unavailable
Children and adolescents living with HIV	“children with HIV” OR “children living with HIV” OR “HIV‐positive children” OR “paediatric HIV” OR “pediatric HIV” OR “HIV‐infected children” OR “HIV‐exposed infant*” OR “HIV‐exposed children” OR “infants with HIV” OR “infants living with HIV” OR “HIV seropositive children” OR “HIV in early childhood” OR “adolescent* with HIV” OR “HIV‐positive adolescent*” OR “HIV‐infected adolescent*” OR “HIV and children” OR “HIV and adolescents”	MeSH: HIV infections; Child; Infant; Adolescent	Title/Abstract	OR within concept; truncation for adolescent* and infant*	Age‐related indexing mapped across databases
Outcomes	“health outcome*” OR “developmental outcome*” OR “well‐being” OR “wellbeing” OR “mental health outcome*” OR “psychosocial support” OR “treatment adherence” OR “child development outcome*” OR “quality of life” OR “family functioning” OR “viral suppression” OR “viral load suppression”	MeSH: Treatment outcome; Mental health; Medication adherence	Title/Abstract	OR within concept; truncation (*) applied	Expanded to capture psychosocial and clinical outcomes
Sub‐Saharan Africa	“sub‐Saharan Africa” OR “SSA” OR “Africa” OR Angola OR Benin OR Botswana OR Burkina Faso OR Burundi OR Cabo Verde OR Cameroon OR Central African Republic OR Chad OR Comoros OR Congo OR “Côte d'Ivoire” OR “Democratic Republic of Congo” OR DRC OR Djibouti OR Equatorial Guinea OR Eritrea OR Eswatini OR Ethiopia OR Gabon OR Gambia OR Ghana OR Guinea OR “Guinea‐Bissau” OR Kenya OR Lesotho OR Liberia OR Madagascar OR Malawi OR Mali OR Mauritania OR Mauritius OR Mozambique OR Namibia OR Niger OR Nigeria OR Rwanda OR “Sao Tome and Principe” OR Senegal OR Seychelles OR Sierra Leone OR Somalia OR South Africa OR South Sudan OR Sudan OR Tanzania OR Togo OR Uganda OR Zambia OR Zimbabwe	MeSH: Africa South of the Sahara	Title/Abstract	OR within concept	Country list used to maximize sensitivity

Search strategies were adapted for each database to account for differences in indexing and functionality, with controlled vocabulary (e.g. MeSH terms such as “HIV Infections,” “Family” and “Patient‐Centered Care”) combined with free‐text keywords using Boolean operators. Truncation and phrase searching were applied where appropriate to capture variations in terminology (e.g. famil*, adolescen*), and searches were primarily conducted in title and abstract fields, with broader searches used where database structures differed.

### 2.3 Stage 3: Selection of Relevant Articles

Two reviewers (TM and ZN) independently screened the titles and abstracts of identified references. Using Rayyan [15], the reviewers recorded reasons for inclusion or exclusion, and removed any further duplicates that had not been automatically detected. The inclusion and exclusion criteria, outlined in Table 2, were applied, and reasons for exclusions were recorded. Discrepancies were resolved through consensus.

**TABLE 2:** Inclusion and exclusion criteria.

Criteria type	Inclusion criteria	Exclusion criteria
Study type	Primary research studies (qualitative, quantitative, mixed methods)	Systematic reviews, scoping reviews, protocols, editorials, commentaries, opinion pieces
Population	Children and adolescents (0−18 years) living with HIV^a^ and their families	Studies focusing only on adult populations not involving children/families
Intervention	Family‐centred care models, approaches or interventions	Studies not addressing care models or family‐centred approaches
Geographic scope	Conducted in sub‐Saharan Africa	Studies conducted outside sub‐Saharan Africa
Language	Published in English (or other languages accessible to the review team, including through Google Translate)	Non‐English articles without available translation
HIV focus	Studies focusing on care for children living with HIV	Studies not focusing on HIV, or focusing only on prevention (e.g. PMTCT^b^)
Publication type	Peer‐reviewed journal articles, unpublished theses or grey literature	Conference abstracts without full papers
Time frame	Published within the defined review period (2000–2025)	Published outside the defined time frame
Data relevance	Sufficient detail and outcome reporting	Insufficient or unclear relevance to family‐centred care

^a^ Human immunodeficiency virus.

^b^ Prevention of mother‐to‐child transmission.

### 2.4 Stage 4: Charting the Data

Data were extracted using a structured Excel framework to capture study and intervention characteristics, including publication details, country, design, demographics, intervention type, core components, family involvement, setting and outcomes such as ART adherence and psychosocial wellbeing. Each article was charted by one reviewer and cross‐checked by another, with discrepancies resolved in team meetings to ensure accuracy and consistency.

### 2.5 Stage 5: Collating, Summarizing and Reporting the Results

Extracted data were synthesized using descriptive statistics for study distribution and characteristics, and thematic analysis [16] to identify key insights and implications for future FCC interventions. Themes were drawn from review questions and expanded based on findings from studies included.

## 3 Results

**FIGURE 1:**
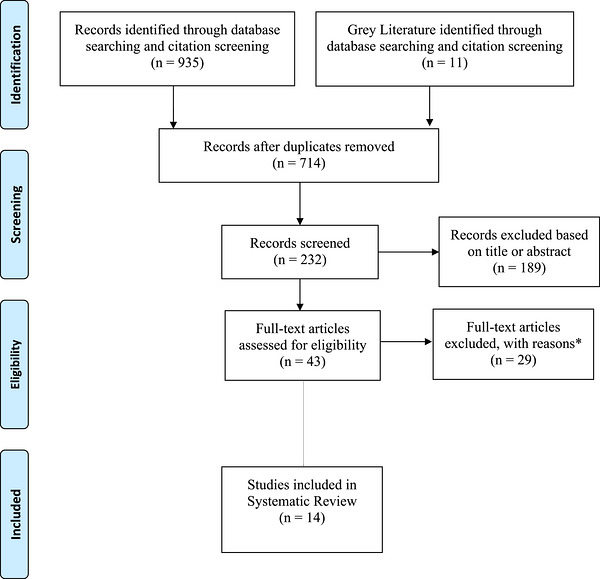
PRISMA flowchart.

Our scoping review yielded a total of 946 references. We identified and removed 714 duplicates, resulting in a total of 232 references included for further screening. The next 189 articles were excluded based on the content of the title or abstract. Forty‐three articles were assessed for eligibility, and 29 were excluded, with reasons (see Figure 1). We describe the characteristics of all included studies (see Table 3), the conceptualization of FCC, the implementation models, and the outcomes and benefits of the FCC model.

**TABLE 3:** Summary of study characteristics.

Citation	Sample size and characteristics	Study design	Study location	Age category	Gender	FCC focus area	Intervention type	Key numeric results
Mavhu et al., 2013	229 children and adolescents, caregivers, healthcare workers and community members	Mixed methods	Zimbabwe	6−18 years: Median age: 14 years	All: 59% female	Psychosocial support	Counselling and peer support	Adherence: 129/229 (67%) 69% female versus 64% male; 76% <13 years versus 69% 13–15 years versus 55% >16 years Depression: 63% were at risk of depression
Achema and Ncama, 2016	16 children, caregivers, nurse practitioners	Qualitative	Nigeria	11−14 years	All	Caregiver involvement	Family‐centred service delivery	N/A
Massavon et al., 2014	1623 children	Retrospective cohort	Uganda	0−18 years	All	Survival and retention	Community and facility‐based FCC	Retention: CHBC: 94.8% versus 84.7%
Gamell et al., 2016	547 children, 200 pregnant women	Mixed methods	Tanzania	3−5 years	All	Integrated service delivery	Bundled HIV services	ART coverage increase: 79.6%−98.1% Lost‐to‐follow‐up rate decrease: 20.4%−10.8%
Van Winghem et al., 2008	648 children, caregivers	Mixed methods	Kenya	<15 years	All	Adherence and literacy	Psychosocial and educational support	N/A
Byakika‐Tusiime et al., 2009	41 children, 124 women	Quantitative	Uganda	<18 years	All	Adherence and mental health	Family treatment model	Mean adherence was over 94% at enrolment but declined over time. Depression significantly associated with incomplete adherence.
Mburu et al., 2014	58 adolescents, parents, healthcare providers	Qualitative	Zambia	10−19 years	All	Social support and stigma reduction	Community‐based FCC	N/A
Luyirika et al., 2013	Children	Observational	Uganda	0−17 years	All	Scale‐up and integration	Integrated FCC model	ART accessibility: 19% of 22,000 patients are children versus 10% national target
Kajubi et al., 2014	35 children	Qualitative	Uganda	8−17 years	All	Communication and disclosure	Therapeutic communication	N/A
Fielder and Kwatampora, 2009	Family—2 parents and 4 children	Case study	Kenya	3 children: one aged 4 and twins aged 5 years	All	Family‐based ART initiation	Staged ART introduction	Pill counts were consistent with a high level of adherence.
Bhana et al., 2014	65 pre‐adolescents and their families	Pilot study	South Africa	10−13 years	All	Mental health and family support	Psychosocial intervention	N/A
Khumalo et al., 2020	25 caregivers, 17 healthcare workers	Qualitative	Eswatini	Caregivers of children: 0–14 years	All	Disclosure and acceptability	Family‐centred model	N/A
Ashburn et al., 2021	379 children, 363 caregivers	RCT (12‐month follow‐up)	Eswatini	0−14 years	All	Viral suppression, retention	Family‐centred HIV care model	Viral suppression at 12 months: FCCM 79.7% versus SOC 69.8%; Undetectable VL: FCCM 78.7% versus SOC 65.7%
Okoko et al., 2020	127 children	Quantitative	Kenya	0−14 years	All	Index testing and identification	Family‐centred index testing	127 (4.5%) had HIV diagnosed, 78.7% linked to care and 91.4% eligible initiated ART. Family testing: 4.5% versus inpatient 1.8% versus outpatient testing 1.6%

### 3.1 Study Characteristics

In this scoping review, we identified 14 studies that explored various aspects of FCC for children and adolescents living with HIV across eight countries in SSA. Uganda contributed the most studies (*n* = 4), followed by Kenya (*n* = 3), Eswatini (*n* = 2) and one study each from South Africa, Zimbabwe, Nigeria, Zambia and Tanzania. The studies were published between 2008 and 2021, reflecting a sustained interest in FCC as a strategy to improve child and adolescent HIV outcomes in the region.

Studies varied considerably in sample size and design, including qualitative approaches (*n* = 5), mixed methods (*n* = 3), quantitative studies (*n* = 4), one retrospective cohort and a randomized controlled trial (RCT) conducted in Eswatini with a 12‐month follow‐up period. Age ranges spanned from infancy to late adolescence, with most studies focusing on children aged 0–14 years, while others included adolescents up to 19 years. Six studies targeted children exclusively, four focused on adolescents, three included both children and caregivers, and one focused on caregivers of children living with HIV. Gender representation was generally inclusive across all the studies.

The FCC interventions addressed diverse focus areas. Psychosocial support, including counselling and peer support, was the most common theme (*n* = 5), followed by adherence and retention in care (*n* = 5). Other areas included caregiver involvement (*n* = 3) and integrated service delivery models (*n* = 4), such as bundled HIV services and family‐based testing. Most interventions combined multiple FCC components, highlighting the holistic nature of these approaches.

### 3.2 Conceptualizing FCC

The conceptualization of FCC within the context of child and adolescent HIV care in SSA reflects a paradigm shift from individualized, biomedical models of service delivery to holistic, relational and community‐embedded approaches. Across the literature reviewed, FCC is consistently framed as a comprehensive strategy that positions the family—not just the child or adolescent—as the central unit of care, support and decision‐making. This reconceptualization is grounded in the recognition that HIV is not experienced in isolation but is deeply embedded within familial, social and economic structures that shape health outcomes and access to care [9, 10, 17, 18, 19, 20, 21].

FCC is conceptualized as a multidimensional framework integrating clinical services with psychosocial support, caregiver empowerment and community engagement [19, 22]. It moves beyond the co‐location of services to promote a continuum of care that is responsive to the needs of all family members affected by HIV. This includes early diagnosis, access to ART, nutritional supplementation, mental health services and treatment literacy [9, 20, 23, 24]. The bundled nature of FCC interventions is seen as essential for improving uptake, retention and adherence to HIV care, particularly in resource‐constrained settings [25].

A key conceptual element of FCC is the emphasis on relational accountability and shared responsibility. Models such as the MTCT‐Plus Family Treatment Model [18] and the VUKA Family Program [24] underscore the importance of involving caregivers—especially parents and guardians—in treatment planning, adherence monitoring and psychosocial support. These models recognize that children's health outcomes are inextricably linked to the wellbeing and engagement of their caregivers [18, 24]. FCC thus reframes care as a collaborative process, where families are not passive recipients but active partners in the management of HIV.

The psychosocial dimension of FCC is another defining feature of its conceptualization. Studies from Zimbabwe [22], Uganda [18, 20, 21, 26] and Zambia [27] highlight the role of FCC in addressing stigma, mental health challenges and communication barriers within families. FCC is seen as a mechanism for facilitating open dialogue about HIV status, treatment and future aspirations—particularly for adolescents who often face silence, secrecy and isolation within their households. This relational and communication aspect of FCC is critical for fostering resilience, self‐efficacy and long‐term engagement in care.

Moreover, FCC is conceptualized as a platform for decentralized and community‐based service delivery. Studies from Kenya [23, 28, 29] and Uganda [18, 20, 21, 26] demonstrate how FCC models can be adapted to primary healthcare settings, thereby enhancing accessibility for rural and underserved populations. This decentralization is supported by task‐shifting strategies, where nurses and community health workers are trained to deliver child and adolescent HIV care, reducing reliance on specialist providers and expanding the reach of FCC interventions.

Increasingly, FCC is also being framed within rights‐based and equity‐oriented paradigms. For an RCT conducted in Eswatini, FCC was conceptualized as a strategy for improving viral suppression and retention in care by addressing structural barriers to access and continuity [9]. By embedding FCC within broader health system reforms and aligning it with global initiatives such as the Global Alliance to End AIDS in Children by 2030 [4], the model is positioned not only as a best practice but as a necessary framework for achieving universal health coverage and child health equity.

The ways in which FCC is conceptualized in the literature we reviewed are both expansive and contextually grounded. It encompasses clinical integration, relational support, decentralized service delivery and rights‐based advocacy. FCC is increasingly promoted as a transformative model that responds to the lived realities of children and adolescents living with HIV, leveraging the strengths of families and communities to support sustainable health outcomes.

### 3.3 Implementation Models of FCC

The implementation of the FCC in child and adolescent HIV services across SSA has taken various forms, reflecting the diversity of health system capacities, socio‐cultural contexts and programmatic priorities. The literature reviewed revealed a spectrum of FCC models ranging from facility‐based integrated care to community‐driven approaches, each with distinct operational features and outcomes.

### 3.4 Facility‐Based Integrated FCC Models

One of the most widely documented implementation strategies involves integrating FCC into existing health facility structures, particularly maternal and child health clinics. These models co‐locate services for children with HIV, their caregivers and other family members, enabling simultaneous access to ART, nutritional support and psychosocial counselling. For example, the MTCT‐Plus Family Treatment Model implemented in Uganda [18], the Kenya comprehensive HIV programme [29] and the Vuka Family Program in South Africa [24] provided ART to both mothers and children, with additional support for mental health and treatment literacy. In Kenya, integrated FCC models embedded within primary healthcare facilities enabled early diagnosis and treatment initiation for children, supported by trained nurses and community health workers [28]. A study conducted in Uganda reported that 19% of 22,000 patients enrolled in an integrated FCC model were children, surpassing the national target of 10%. In Tanzania, there was an increase in ART coverage from 79.6% to 98.1% and a reduction in loss‐to‐follow‐up from 20.4% to 10.8% following implementation of a bundled HIV service model. These findings suggest that integrated FCC models may improve retention and adherence, particularly when caregivers receive concurrent treatment.

### 3.5 Community‐Based FCC Models

Community‐based FCC models extend care beyond clinical settings, leveraging community health workers, peer support networks and home‐based services to reach families in rural and underserved areas. These models are particularly effective in contexts where access to health facilities is limited or where stigma and fear of disclosure hinder clinic attendance. In Zambia, Mburu and colleagues described a social‐ecological approach involving community mobilization and household‐level counselling, resulting in improved health outcomes for both children with HIV and their caregivers [27]. Community‐based models often incorporate task‐shifting strategies, training lay health workers to deliver HIV services and monitor adherence. This decentralization expands service coverage and fosters trust and engagement within communities.

### 3.6 Hybrid and Multicomponent FCC Models

Several programmes have adopted hybrid models that combine facility‐based and community‐based elements. These multicomponent approaches aim to provide continuity of care across different settings and life stages. The VUKA Family Program in South Africa [24] offers structured psychosocial interventions for early adolescents and their caregivers through both clinic‐based sessions and community follow‐up. The programme addressed mental health, stigma and communication within families, contributing to improved ART adherence and emotional wellbeing.

In Eswatini, an RCT evaluating a structured FCC model over 12 months highlighted that children enrolled in the FCC model achieved viral suppression rates of 79.7% compared to 69.8% in standard care, and undetectable viral load rates of 78.7% versus 65.7%, suggesting clinical benefits from comprehensive family engagement [9]. The intervention included caregiver counselling, family‐based treatment planning and regular follow‐up visits, demonstrating significant improvements in clinical outcomes compared to standard care.

### 3.7 Outcomes and Benefits of FCC

The implementation of FCC models in SSA has yielded a range of positive outcomes for children and adolescents living with HIV, as well as for their caregivers and broader family units. We found in the literature reviewed that FCC contributes consistently to improved clinical, psychosocial and programmatic outcomes.

One of the most frequently reported benefits of FCC is enhanced ART adherence and retention in care. Studies from Uganda [18, 20, 21], Kenya [23, 28, 29] and Eswatini [9, 10] show that when caregivers are actively involved in treatment planning and monitoring, children are more likely to remain engaged in care and adhere to their medication regimens. For instance, a study in Uganda observed mean adherence rates exceeding 94% at enrolment, although adherence declined over time, with depression significantly associated with incomplete adherence [18]. Mavhu and colleagues in Zimbabwe reported an overall adherence rate of 67%, with higher adherence among females (69%) compared to males (64%) and among younger children (76% for <13 years) compared to older adolescents (55% for >16 years) [22]. These findings may underscore the importance of psychosocial support and age‐sensitive interventions.

Retention outcomes were also notable. A study in Uganda documented retention rates of 94.8% in community home‐based care compared to 84.7% in facility‐based care [21]. This may highlight the effectiveness of decentralized models. Integrated service delivery in Tanzania further improved ART coverage from 79.6% to 98.1% and reduced loss‐to‐follow‐up from 20.4% to 10.8% [25]. These results may illustrate the programmatic advantages of bundled services.

FCC also contributes to early diagnosis and timely initiation of treatment, particularly through family‐based index testing approaches. In Kenya, Okoko and colleagues reported that family‐centred index testing identified HIV in 4.5% of children tested, compared to 1.8% in inpatient testing and 1.6% in outpatient testing. Among those diagnosed, 78.7% were successfully linked to care, and 91.4% initiated ART [28]. This may demonstrate the effectiveness of family‐based strategies for case finding and linkage. By testing all family members of individuals known to be living with HIV, programmes may have been able to locate people who had acquired HIV at earlier stages, initiate ART promptly and reduce HIV‐related morbidity and mortality.

Psychosocial outcomes were equally significant. Studies from Zimbabwe [22], South Africa [24] and Zambia [27] highlight how FCC interventions—especially those incorporating counselling, peer support and family dialogue—reduce stigma, improve mental health and foster resilience among children and adolescents living with HIV. For example, a study in Zimbabwe found that 63% of adolescents were at risk of depression. This may reinforce the need for integrated psychosocial support within FCC models. The VUKA Family Program, described above, may have addressed these challenges through structured interventions, contributing to improved emotional wellbeing and adherence among early adolescents [24].

FCC models also enhance service uptake and continuity by reducing logistical and financial barriers to care. Integrated service delivery models, such as those implemented in Uganda [18, 26] and Kenya [29], allow families to access multiple services—ART, nutritional support and mental healthcare—at a single location, thereby improving efficiency and reducing missed appointments. This bundling of services is particularly beneficial in rural and resource‐constrained settings, where transportation costs and clinic fragmentation often hinder access.

Furthermore, the FCC has been shown to strengthen health system responsiveness and equity. By decentralizing care and incorporating task‐shifting strategies, FCC models extend services to underserved populations and reduce dependence on specialist providers. In Zambia [27] and Uganda [20], training nurses and community health workers to deliver child and adolescent HIV care within FCC frameworks expanded coverage and improved outcomes without compromising quality.

## 4 Discussion

SSA continues to bear the brunt of the global child and adolescent HIV epidemic, with children and adolescents facing disproportionate challenges compared to many adults in accessing and sustaining HIV care. Despite notable progress in PMTCT and the expansion of ART, significant gaps remain in diagnosis, treatment adherence and psychosocial support for young populations. Our review highlights that these gaps are not only clinical but embedded in the social and familial contexts in which children and adolescents live. FCC emerges as a critical and contextually appropriate response to these challenges, offering a holistic framework that integrates clinical, psychosocial and relational dimensions of care.

The conceptualization of FCC across the studies reviewed reflects a paradigm shift from individualized biomedical models to relational and community‐embedded approaches. FCC is consistently framed as a multidimensional strategy that positions the family—not just the child—as the central unit of care and decision‐making. This reconceptualization acknowledges that HIV is experienced within complex family systems and social structures, which shape health outcomes and access to care. For example, models such as MTCT‐Plus in Uganda [18] and the VUKA Family Program in South Africa [24] operationalized this principle by combining ART provision with psychosocial support and treatment literacy. These interventions appear to have demonstrated measurable improvements in adherence and retention, and may validate the conceptual argument that FCC must address relational and emotional needs alongside clinical care.

The conceptual emphasis on integration and decentralization within FCC frameworks may reflect a deliberate attempt to overcome structural barriers in child and adolescent HIV care. Integrated facility‐based models, which co‐locate services and bundle clinical and psychosocial support, appear to illustrate how FCC operationalizes the principle of holistic care. These models seem to do more than improve convenience; they appear to address systemic fragmentation that often leads to missed appointments and treatment interruptions. By embedding ART provision alongside nutritional and mental health services, integrated FCC models could create a single point of care that reduces logistical burdens for families and strengthens continuity of treatment.

Decentralization, another core conceptual theme, can be seen to extend this logic by shifting care closer to households and communities. Community‐based FCC models seem to demonstrate that moving services beyond facility walls is not merely a logistical adjustment but a transformative approach that fosters trust, reduces stigma and enhances engagement. These models leverage task‐shifting strategies and community health workers to deliver care in familiar environments, thereby mitigating barriers such as transportation costs and fear of disclosure.

The psychosocial dimension of FCC is not only an adjunct to clinical interventions; it is a foundational component that shapes adherence, resilience and long‐term engagement in care. Evidence from Zimbabwe [22] and Zambia [27] may underscore that psychosocial challenges—such as depression risk and stigma—remain pervasive among children and adolescents living with HIV. These findings suggest that FCC models must go beyond clinical co‐location to actively address mental health vulnerabilities and relational dynamics within families.

Our findings also highlight the importance of understanding family dynamics and gendered caregiving roles in shaping the effectiveness of FCC interventions. Across studies, caregiving responsibilities were predominantly carried by mothers and female relatives, while fathers were often minimally engaged or absent from programme descriptions. In many settings, grandparents, older siblings and other extended kin played critical roles in supporting treatment adherence and psychosocial wellbeing, reflecting the distributed nature of caregiving in many African households. These dynamics have important implications for FCC design, suggesting that interventions must move beyond assumptions of nuclear families and actively engage diverse caregiving networks to maximize impact.

The high proportion of adolescents at risk of depression reported in Zimbabwe suggest a critical gap in child and adolescent HIV programming: the absence of structured psychosocial support within routine care. This may reinforce the conceptual argument that the FCC should integrate counselling and family dialogue as core elements rather than optional add‐ons. Similarly, the success of the VUKA Family Program could be seen to illustrate how structured interventions targeting stigma and communication can transform family relationships, thereby improving adherence and emotional wellbeing. These outcomes suggest that psychosocial interventions embedded within FCC frameworks are not only feasible but essential for sustaining treatment outcomes. Community‐based FCC models, such as those documented in Zambia, further seem to demonstrate the value of addressing stigma at the household and community level. By fostering open communication and reducing secrecy around HIV status, these models appear to create enabling environments for adolescents to develop health literacy and self‐management skills.

The adaptability of FCC across diverse contexts underscores its potential as a scalable strategy for child and adolescent HIV care. Evidence from Eswatini [9] and Uganda [18, 21] appears to demonstrate that FCC models can deliver strong clinical outcomes when tailored to local health system structures and family dynamics. The success of structured interventions in Eswatini, for example, seems to reflect how embedding caregiver counselling and family‐based treatment planning within routine care can strengthen viral suppression and retention. These findings suggest that FCC is not limited to psychosocial benefits but can directly influence biomedical outcomes when implemented comprehensively.

Equally important is the interplay between clinical and psychosocial dimensions observed in Uganda [26]. High initial adherence rates, followed by declines linked to depression, may be seen to highlight the fragility of treatment success in the absence of mental health support. This reinforces the conceptual argument that the FCC must integrate psychosocial interventions as core components rather than optional enhancements. Models that combine adherence monitoring with mental health screening and caregiver engagement may be better positioned to sustain long‐term outcomes. These insights can illustrate that FCC's adaptability lies in its ability to respond to structural and relational determinants of health.

Despite these promising findings, several critical gaps remain in the literature on FCC in child and adolescent HIV programming in SSA. First, there is a notable lack of longitudinal research that tracks the sustained impact of FCC on child health outcomes, psychosocial development and family dynamics over time. Most studies rely on cross‐sectional or short‐term evaluations, limiting the understanding of FCC's long‐term effectiveness and scalability. Second, much of the literature implicitly assumes a nuclear family model, thereby overlooking the roles of extended family members and non‐biological guardians who are often central to caregiving in African contexts. This narrow framing risks excluding key actors from FCC interventions and underestimating the complexity of family structures.

Third, male caregivers—including fathers—are frequently underrepresented in FCC conceptualization, research and programmatic design, despite emerging evidence that their involvement can positively influence child health outcomes. Fourth, there is currently no universally accepted framework for FCC in child and adolescent HIV care, resulting in inconsistencies in how FCC is defined, operationalized and evaluated across studies and settings. This lack of standardization poses challenges for comparative analysis and evidence synthesis.

Fifth, limited evidence exists on the cost‐effectiveness and feasibility of FCC at scale. Most documented interventions are donor‐funded pilot projects, and few studies assess the financial sustainability or integration of FCC into national health systems. Sixth, across the included studies, no negative clinical or psychosocial outcomes related to FCC were reported. While several studies described implementation barriers (e.g. resource constraints, stigma, caregiver burden), none documented adverse effects of FCC interventions on children, adolescents or caregivers. The absence of reported negative outcomes should be interpreted cautiously, as it may reflect limitations in study designs or reporting practices rather than the absence of challenges. Finally, there are a few FCC models specifically tailored to adolescent caregivers and their children—a growing demographic in regions heavily affected by HIV. These gaps highlight the need for more inclusive, context‐sensitive and longitudinal research to inform the next generation of FCC interventions.

This review has several limitations. First, we included only studies published in English, which may have excluded relevant research conducted or reported in other languages. Second, although we searched grey literature sources, some programme reports and unpublished evaluations may not have been accessible, potentially introducing publication bias. Third, the heterogeneity in how FCC was defined and operationalized across studies limited comparability. Fourth, most included studies were small‐scale or pilot interventions, which may not reflect implementation at scale. Finally, as a scoping review, we did not formally assess study quality [12].

## 5 Conclusions

Our scoping review highlights FCC as a promising and transformative approach to child and adolescent HIV care in SSA. The available evidence suggests that FCC improves treatment adherence, retention in care, psychosocial wellbeing and service integration; however, its implementation remains inconsistent and conceptually fragmented. The lack of standardized frameworks, limited longitudinal data and underrepresentation of diverse caregiving structures—particularly male and adolescent caregivers—underscores the need for inclusive, context‐sensitive research. To achieve meaningful impact, FCC may have to transition from small‐scale pilots to scalable, sustainable components of national HIV strategies, rooted in the lived realities of families and communities most affected by the epidemic.

## Author Contributions

**Tawanda Makusha and Zinhle Ngubo**: conceptualization of scoping review, screening, analysis, writing – original draft, writing, review and editing. **Tawanda Makusha**: writing, review and editing. **Tawanda Makusha and Zinhle Ngubo**: adjudication of conflicts in screening, writing, review and editing. **Moherndran Archary**: conceptualization of project, writing, review and editing. **Janet Seeley**: conceptualization of project and scoping review, writing, review and editing. All authors read and approved the final version of the manuscript.

## Funding

TM and MA are supported by funding from the National Institute for Health Research Global Health Research Professorship Award NIHR303161. The Africa Health Research Institute is supported by core funding from the Wellcome Trust (grant number 201433/Z/16/A).

## Conflicts of Interest

The authors declare that they have no conflicts of interest.

## Disclaimer

The views expressed are those of the authors and not necessarily those of the NIHR, the Department of Health and Social Care or the UK Government.

## Data Availability

All relevant data are included in the paper.
